# Follow-Up Treatment After Inpatient Therapy of Patients With Unipolar Depression—Compliance With the Guidelines?

**DOI:** 10.3389/fpsyt.2020.00796

**Published:** 2020-08-07

**Authors:** Lukas Weiß, Almut Zeeck, Edit Rottler, Heinz Weiß, Armin Hartmann, Jörn von Wietersheim, The INDDEP Study Group

**Affiliations:** ^1^ Department of Psychosomatic Medicine and Psychotherapy, University Medical Center, Ulm, Germany; ^2^ Department of Psychosomatic Medicine and Psychotherapy, Medical University Hospital, Freiburg, Germany; ^3^ Department of Psychosomatic Medicine and Psychotherapy, Robert-Bosch-Krankenhaus, Stuttgart, Germany

**Keywords:** major depression, treatment guideline, compliance with guideline, antidepressants, psychotherapy

## Abstract

**Objective:**

To date, there is only a limited number of studies evaluating the implementation and effects of treatment guidelines. Therefore, this study aimed to determine how many patients diagnosed with a major depression were treated in compliance with the German treatment guideline after hospital treatment, and whether a deviation from the guideline resulted in a less favorable development.

**Methods:**

Five hundred two patients, which originally participated in the INDDEP-study, were included. Data were collected at admission and discharge from eight different psychosomatic (psychotherapeutic) hospitals in Germany as well as 3 months and 1 year after hospital treatment. Data on depressive symptomatology were assessed by QIDS-C (clinical interviews). By phone interviews, the clinical course and the outpatient treatments were assessed. Statistical analyses compared patients who were treated in compliance with the German treatment guideline with those who were not.

**Results:**

Seventy-nine point one percent of the outpatient treatments complied with the treatment guideline. Eleven point eight percent of the patients were treated with medication only, 60.2% with psychotherapy only, and 28.0% with a combination. There was no difference in the clinical outcome (depression) with regard to guideline compliance. Cases in which deviation from the guideline occurred (20.9%) were younger and had a less severe depressive symptomatology at admission and after hospital treatment.

**Conclusion:**

After treatment in a psychosomatic hospital or day hospital, the majority of patients with a depressive disorder received adjacent treatment in accordance with the German guideline and with a clear focus on psychotherapy. Deviations from the guideline did not result in a less favorable course of the illness.

**Clinical Trial Registration:**

ISRCTN20317064, retrospectively registered 31.07.2012

## Introduction

An important objective of health care policies as well as national and international medical associations is to provide as many patients as possible with state-of-the-art treatments. Therefore, the results of evidence-based medicine are summarized in treatment guidelines to provide physicians and therapists with compact information about the latest findings on etiology, epidemiology, diagnostics and treatments for specific clinical diagnoses.

The results of scientific studies, clinical experience, and discussions within in the professional associations lay the foundation for these guidelines, which increasingly influence the clinical practices of physicians and therapists (1). What is rarely done, but needed, are studies that evaluate the implementation of guidelines in clinical practices as well as the specific effects of a therapy in compliance with a guideline on the outcome of patients. Thus, the questions remain, in how far the requirements of the guidelines are implemented and if the patients benefit from these implementations. Today, evidence-based means that the recommendations in the guidelines are justified by the results of empirical studies. Some research demands that guidelines should not receive the addendum “evidence-based” until studies have been able to show a positive effect on the outcome of patients treated in accordance with the guideline (2).

It is difficult to predict the effect of full guideline compliance on the outcome, since most of the guidelines are complex and pertain to different modalities such as medication, psychotherapy, and their combination. It is assumed that treatment in compliance with a guideline has a more favorable effect on the patient’s disease than a treatment that is not in compliance with a guideline. A frequent question is whether an individual treatment at the discretion of the therapist (and the patient him- or herself) would produce equally good results. In addition, the resources to follow recommendations must exist as well. For depressive patients, for example, the recommendation of an outpatient psychotherapy cannot be implemented if there are no therapy options or if the waiting times are too long.

So far, no or only slightly positive effects could be found for the treatment of mental disorders on the basis of a guideline. In a systematic review, 18 studies on guidelines for different mental diagnoses, among them nine studies on depression or affective disorders, were analyzed. The authors conclude that there is insufficient evidence to draw firm conclusions on the effects of implementation of specific psychiatric guidelines (3). In a Cochrane Review, the effect on guideline implementation for patients with psychotic disorders was studied. The authors identified five studies and report methodological problems and only uncertain effects of guideline implementations (4). Other studies examined how to better implement guideline requirements in medical practice (5, 6). They identified basic requirements that a medical guideline should meet to be used in practice. For example, easy-to-read tables and training course to inform therapists and patients should be applied.

There are only few publications regarding the effects of guideline implementations on the therapy outcome of patients with a depressive disorder. A study in England concluded that special physician training on the treatment of depression resulted neither in a better identification of patients nor improved progressions of the illness (7). In a large British project (IAPT), a short-term guideline psychotherapy was developed and evaluated. For this purpose, 3,600 therapists were hired and trained (8, 9) with the effect that the therapy had good effects and more people could gain access to these treatments. However, the extent of these results varied between the participating centers.

To date, however, there are no studies assessing the effects of compliance with the guidelines on the outcome of depressive patients in the follow-up of an inpatient or day-clinic treatment. A reason in this case may be, that the requirements of the current guidelines are quite general, and another is that the treatments must partially be organized by the patient him- or herself (e.g. in the case of psychotherapy). Furthermore, the treatment, such as psychotherapy, must be offered by the health care system.

### Current Guideline Requirements for Treatment Following Inpatient Therapy

Internationally, some evidence-based guidelines for the treatment of a major depression exist. Their recommendations are often based on the same studies and are therefore, as expected, quite similar (10–12). The treatment options may, however, differ between different countries, for example, in terms of the availability of inpatient psychotherapeutic treatment or outpatient psychotherapy. In general, therapeutic guidelines should be evidence-based and refer to the results of existing studies. If no study results exist, they should be based on clinical recommendations. However, this leads to some uncertainties. Since no precise study results indicate that one antidepressant group (e.g. SSRI) yields better results than the other (e.g. Tricyclic antidepressants), the recommendation states only to “take an antidepressant in the recommended dose” but the drug itself is not further specified. Related to psychotherapy, the duration or the specific psychotherapeutic method (e.g. cognitive behavior therapy or interpersonal psychotherapy) is not specified because there is not enough scientific evidence to recommend a specific method or the necessary duration of the therapy (13).

The German Guideline for the Treatment of Unipolar Depression is an evidence-based S3 guideline. It was jointly developed by several scientific professional associations and published in 2015 under the leadership of the “Deutsche Gesellschaft für Psychiatrie und Psychotherapie, Psychosomatik und Nervenheilkunde” (DGPPN) [German Association of Psychiatry and Psychotherapy, Psychosomatics and Neurology] (13).

The guideline states some recommendations for treatments following inpatient or day-hospital treatment. It differentiates between acute therapy (as an intensive, sometimes initial measure for the reduction of the symptoms) and maintenance therapy to maintain an improvement. Inpatient treatment was set equal to an acute treatment, since it is only permitted if the symptoms are sufficiently severe and if an outpatient treatment would be insufficient. Next, the following recommendations can be concluded for post-inpatient treatment of major depressive disorders:

1. Outpatient continuation of psychotherapy for at least 8 to 12 months at a lower frequency of sessions (than in the acute treatment).
2. Outpatient continuation of the anti-depressive medication: In the case of a first depressive episode the treatment with antidepressants should continue in the same dose as during the acute therapy over a period of at least 4 to 9 months. In the case of a recurrent, chronic, or chronic-recurrent depression, medication should be taken for at least 2 years in the same dose.
3. When psychotherapy is combined with anti-depressants during the acute treatment (hospital treatment), the follow-up treatment should also consist of a combined therapy.

The objective of our study was to evaluate data from the naturalistic and multi-centric INDDEP study (“**IN**patient and **D**ay clinic treatment of **DEP**ression”)—an observational study of inpatient and day-clinic treatment of patients with a depressive disorder in psychosomatic hospitals—with regard to guideline implementation. The purpose of the INDDEP study was to analyze the effect of acute treatments of unipolar depression in an inpatient or day-clinic setting. After the acute treatment, the follow-up treatment of patients was examined for a period of 1 year. The main intent of the INDDEP study was to identify prognostic and prescriptive predictors for the hospital and day patient treatment. It was found that at admission, patients treated in an inpatient setting were more depressed than day-clinic patients. In both settings, patients were able to achieve a significant symptom reduction, and there was no significant difference in terms of the clinical outcome between both settings (14, 15).

The following aspects of the INDDEP study were particularly suitable to assess guideline implementation: The multi-centric approach with eight centers that can be considered representative for psychosomatic hospitals, the large number of study participants, the naturalistic study design as well as two follow-up assessments during a period of 1 year, which documented the treatments patients received after their discharge. This made it possible to evaluate the implementation of the requirements of the German Guideline on Unipolar Depression in the outpatient phase that followed discharge. For this substudy, the following research questions were developed:

1. How many patients were treated in accordance with the requirements of the Guideline on Unipolar Depression in the first year after discharge from an inpatient or day-clinic treatment?
2. Are there differences in terms of sociodemographic and disease-specific characteristics between the patient groups that were treated in the follow-up therapy either in compliance or not in compliance with the guideline?
3. Did patients who were treated in compliance with the guideline have a better clinical outcome?
4. Which form of therapy, outpatient psychotherapy, antidepressant medication or the combination of these two plays the most important role in practice for the decision that the treatment is in accordance with the guideline?

Our main hypothesis was, that patients treated in accordance to the German S3-guideline, have a more favorable course of the depression than those, who are not treated in accordance to the guideline.

## Methods

### Study Design

The study is based on data from the multi-centric and naturalistic INDDEP study (“**IN**patient and **D**ay clinic treatment of **DEP**ression”, trial registration number: ISRCTN20317064). In the INDDEP study, patients diagnosed with unipolar depression were included into the study during an inpatient or day-clinic treatment. The average treatment length was 10.0 weeks (SD=4.3 weeks). For the diagnosis, structured clinical interviews for DSM IV (SKID I and II) were used at patients’ admission (16, 17). Assessment points were admission to the hospital (T0), discharge (T1), and the 3-month (T2) and 12-month (T3) follow up-examinations. The main study results have already been published (14, 15, 18). The study was approved by the Ethics Committee of the University of Freiburg (no. 39/11) and the Ethics Committee of the University of Ulm in April 2011 under the application number 83/11.

### Study Centers

A total of eight centers participated in the INDDEP study including three university hospitals and five non-university institutions. The admission and discharge examinations were performed in the centers; the follow up-examinations were centrally organized by the study centers Freiburg and Ulm and performed by trained research assistants.

### Sample

Inclusion criteria for the study [see study protocol: (19)] were the presence of a unipolar depressive episode (according to DSM IV criteria) as main diagnosis, with a QIDS-C score (Quick Inventory of Depressive Symptoms, clinical rating) higher than 10 (corresponds to a moderate MDE), age between 21 and 65 years, an adequate command of the German language and the consent to the participation in the study. Exclusion criteria were a current or earlier psychotic disorder, a bipolar disorder, substance dependence or current thoughts of suicide. Patients with anti-social personality disorders and cognitive impairment were excluded as well.

In the recruitment phase from September 2011 until April 2014, 604 patients were included. This substudy is on guideline compliance of the follow-up therapies. Thus, information on the treatments in the follow-up period, which were gathered at the follow-up examinations, was obligatory and applicable for 502 patients.

A comparison of the sample with data on the follow-up treatments and the data of patients, who had to be omitted, showed that patients who could not be included were younger (M 38.4 vs. 44.1 years, SD 11.2 vs. 11.6, t=4.5, df= 602, p<0.001), more frequently single (48% vs. 31%; Chi^2^ 14.3; df=5, p = 0.014), and more often unemployed (19.6% vs. 11%; Chi^2^ 9,2; df=3, p=0.026). Their QIDS depression scores were slightly higher at hospital admission (16.8 vs. 15.3, SD 3.0 vs. 3.1; t=4,5; df=602, p<0.001). **Table 1** provides the sociodemographic data of the sample of the guideline substudy.

**Table 1:** Sociodemographic data of the sample (N = 502).

Variables	M	SD	n	%
Age	44	11.6		
Gender (female)			328	65.8
Length of schooling (12–13 years)			256	51.0
Existing partnership			239	47.6
Number of prior depressive episodes	3.3	6.7		
Patients with first depressive episode			141	28.1
QIDS-C-Score (T0)	15.3	3.0		

### Implementation and Assessment Instruments

The study was carried out at eight German study sites. All departments or hospitals provided a treatment program according to the standards for hospitals of psychosomatic medicine in Germany (20). The programs provided time-limited, intense multimodal psychotherapy, including individual psychotherapy sessions (one or two sessions per week), different kinds of group psychotherapy (psychodynamic or symptom oriented), art therapy, music therapy, and body-oriented therapy as well as family sessions. Additional support was provided by a social worker, relaxation groups, sessions with the nursing team, educational elements, and medical care. The use of antidepressants was an optional component of the anti-depressive therapy and was governed by the requirements of the guidelines.

Data for each patient was collected at four specific time-points. T0 was the date of the hospital admission and the start of the acute therapy. The next assessment was the discharge from the hospital (T1). To assess the compliance with the treatment guideline, the follow up-examinations were important. They took place 3 months (T2) and 12 months (T3) after the hospital discharge. At these time points, semi-standardized phone interviews were conducted to assess the patients’ current symptoms, the course of the disease, and the treatments they received after the last assessment. Furthermore, during the follow up-examinations, patients completed questionnaires on their symptoms, their psychological wellbeing and their treatments.

For this study, the following data were included:

1. The admission form provided basic documentation on the socioeconomic data, information about the course of the disease and treatment before admission. Other existing mental and somatic diagnoses were documented as well.
2. At T2 and T3, the Longitudinal Interval Follow-Up Interview [LIFE, (21)] was conducted. Here, the current symptom severity was assessed for each week of the follow up-period. Additionally, information regarding the type and dosage of medication as well as the type and duration of psychotherapy was collected.
  All telephone interviews were performed by two trained research assistants, with a master degree in psychology. They received a minimum of two trainings with a duration of 2 days. The research assistants had continuous contacts to discuss problematic interviews and their ratings. They also were trained in performing the QIDS-C and the Social and Occupational Functioning Assessment Scale (SOFAS). The interrater reliability between the two raters was checked during their trainings, but not further during the study.
3. The central outcome parameter of the INDDEP study is the QIDS-C score, which measures the severity of the depression. The QIDS is a 16-item version of the Inventory of Depressive Symptomatology (IDS) that shows high correlations with other instruments for the measurement of depressive symptomatology. It is an internationally used license-free instrument with good psychometric properties. The total score ranges from 0 to 27. Cronbach’s alpha was 0.85 for the QIDS-C (22). The internal consistency in this study (across all assessment points) was 0.81.
4. The SOFAS score was determined for each of the four measurement points. On a rating scale from 0–100, the social functioning level of a was assessed for 2 time periods: the last 4 weeks (in the follow up-examinations) and the last year before admission into the hospital (23).

### Compliance With the Guideline

Compliance with the guideline is assumed if patients are treated continuously either with an anti-depressive drug (in a therapeutic dose) or with psychotherapy or a combination of both. The drug treatment depends also on the drug treatment received in the hospital (for details see *Introduction*). If a first episode of a depressive disorder was diagnosed during admission and the patient was treated with medication, the guideline states that the medication should not be reduced until at least 4 months after discharge. In the case of a recurrent or chronic depression, patients should receive drug treatment for 2 years (exceeding the follow up-period) and may additionally receive psychotherapy.

Only antidepressants including the following substances in a therapeutic dose were considered as guideline-compliant medication: Selective serotonin reuptake inhibitors (SSRI) such as Escitalopram, Citalopram, Sertraline, Fluoxetine, Fluvoxamine and Paroxetine, serotonin, and norepinephrine reuptake inhibitors (SSNRI) such as Venlafaxine and Duloxetine, the Alpha-2 antagonist Mirtazapine, and the group of tricyclic and tetracyclic antidepressants such as Clomipramine, Trimipramine, Opipramol, Amitriptyline, Doxepin, and Nortriptyline. In addition, the serotonin norepinephrine dopamine reuptake inhibitor (SNDRI) Bupropion, monoamine oxidase inhibitors (MAO-I), Agomelatine, an agonist on melatonin-1- and -2-receptors as well as an agonist on 5HT-2C-receptors and the non-classifiable substances Trazodone and Tianeptine were considered part of the anti-depressant group. The therapeutic dosing of each drug was obtained from the “Rote Liste” 2015 [list of all drugs which can be prescribed in Germany (24)].

### Statistics

Data were obtained from the central MS Access file of the INDDEP study. The statistical analyses were performed with the statistics program SPSS (Version 23). The normal distribution was checked with the Kolmogorov-Smirnov test; most of the variables were not normally distributed. Therefore, non-parametric tests were used for the comparisons at one assessment point. Depending on the scale level, group comparisons were calculated with the Mann-Whitney-U test, the Kruskal-Wallis-H test, and the Chi-squared test (exact test according to Fisher). For the evaluation with the QIDS-C, a repeated measures ANOVA was computed. The significance level was p < 0.05. Due to the explorative character of the study, an alpha adjustment was not performed.

## Results

### Treatments at Follow Up-Examinations

At 3-month follow-up (T2) 205 (40.8%) of the patients reported to take an antidepressant medication (SSSRI: 31.3%, tricyclic antidepressant: 16.5%, SSNRI: 15.5%, combination of at least two antidepressants: 29.6%).

At 12-month follow-up (T3) 266 (53%) of the patients took an antidepressant (SSRI: 36.5%, tricyclic antidepressant: 12.4%, SSNRI: 18.8%, combination of at least two antidepressants 27.1%).

At T2, 367 patients (73.1%) underwent psychotherapeutic outpatient treatments (cognitive-behavioral therapy: 21.3%, psychodynamic therapy: 38.2%, psychoanalytic treatment 9.4%, other forms: 12.9%).

At T3, 401 patients (79.9%) underwent psychotherapy. The distribution of psychotherapeutic methods was similar to those at T2.

### Guideline Compliance of the Follow-Up Treatment

The treatment complies with the guideline, if psychotherapy, medication, or a combination of both is provided (see *Methods*).

To assess the criterion “compliance of the guideline by psychotherapy”, so called “outpatient psychotherapy treatment groups” were defined. The respective data was collected in the interviews at T2 and T3. This resulted in four groups that show if and how long psychotherapy was provided after the hospital treatment (see **Table 2**). Using this criterion, almost 70% of the treatments comply with the guideline; another 10% of the patients received psychotherapy between the 3-month and the 1-year follow up-assessment.

**Table 2:** Follow-up treatment groups according to psychotherapy (N = 502).

Psychotherapy Treatment Group	n	%
No continuous outpatient psychotherapy (from T1 to T3)	84	16.7
Continuous outpatient psychotherapy (from T1 to T3)	350	69.7
Outpatient psychotherapy from T1 to T2	17	3.4
Outpatient psychotherapy from T2 to T3	51	10.2

T1: Discharge, T2: 3-month follow-up, T3: 1-year follow-up.

To assess the criterion “compliance with the guideline by medication”, “medication treatment groups” were formed on the basis of the medication data from T1, T2, and T3 (see **Table 3**). Of greatest interest is whether and when a prescribed medication was discontinued after the hospital stay. The treatment of at least 30% of the patients complied with the guideline (somewhat more patients with a first episode and therapy for at least 4 months), but also, that in some cases a medication was discontinued early (approx. 15%) and a new medication started (approx. 15%).

**Table 3:** Follow-up treatment groups according to medication (N = 502).

Medication treatment group	n	%
No continuous guideline-compliant medication (at T1, T2, and T3)	166	33.0
Continuous guideline-compliant medication (at T1, T2, and T3)	152	30.3
Discontinuation of the guideline-compliant medication prior to week 18 (medication at T1 and T2)	73	14.5
Discontinuation of the guideline-compliant medication after week 18 (medication at T1 and T2)	23	4.6
Start of a guideline-compliant medication after T1	76	15.1
Guideline-compliant medication at T1, then discontinuation and restart after T2	12	2.4

T1: Discharge, T2: 3-month follow-up, T3: 1-year follow-up.

A combination of the criteria medication and psychotherapy shows that 79.1% (n = 397) of all patients were treated in compliance with the guideline in the follow-up period of 1 year. For 20.9% (n = 105) of the patients, the therapy did not comply with the guideline (e.g. no psychotherapy, no follow-up medication, early reduction of the medication dose).

### Comparison of the Groups According to Compliance With the Guideline

The two groups (compliance with the guideline vs. no compliance with the guideline) were compared with regard to sociodemographic and clinical variables. The items gender, nationality, civil status, children of their own, children in the household, schooling, professional training, occupation, and employment did not show any significant differences in their distribution between the two groups. The analyses of disease-related variables such as the number of prior episodes, the length of the current episode, double depression, depression state, i.e. acute, chronic, recurrent or chronic recurrent, the number of additional somatic diagnoses and personality disorders did not differ significantly either.

There were a few significant differences between patients who were treated in compliance with the guideline and those who were not. These are summarized in **Table 4**.

**Table 4:** Differences between the patient groups treated in compliance with the guideline and not in compliance with the guideline (N = 502).

Variable	In compliance with the guideline (N=397)		Not in compliance with the guideline (N=105)		Test statistic		p (2-sided)
	M	SD	M	SD	U	z	
Age	44.8	12.8	41.3	12.7	17522	2,5	0.012
Age at disorder onset	34.0	13.7	30.9	13.1	17715	2,2	0.028
Social Functioning SOFAS (T0)	49.44	9.11	52.33	10.19	17315	2,7	0.008
Social Functioning SOFAS (T3)	64.38	13.58	68.08	14.40	17452	2,5	0.014
Comorbidity (T0) Number of additional axis I diagnoses	1.06	1.13	0.90	1.18	18912	1,5	0.123
Number of somatic diagnoses	1.09	1.38	1.19	1.49	20308	0,4	0.664
	**In compliance with the guideline**		**Not in compliance with the guideline**		**Test statistic, df = 1**		**p (2-sided)**
	**%**	**N**	**%**	**n**	**Chi^2^**		
Unable to work prior to being admitted	69.3	266	58.4	59	4,3		0.043
Inpatient treatment (not in the day clinic)	52.9	210	41.0	43	4,7		0.037
Visited a specialist (all specializations) during the follow-up period	72,2	287	53.3	56	13,8		<0.000
Rehospitalization and psychosomatic treatment during the follow-up period (relapse)	9.6	38	1.9	2	6,7		0.008
Treated by an alternative practitioner	9.3	37	2.9	3	4,7		0.026
Attended a support group	7.8	31	1.9	2	4,7		0.027

In general, patients who were not treated in compliance with the guideline were a little bit younger (41.3 vs. 44.8; p=0.12), they were also younger at the onset of the illness (30.9 vs. 34; p=0.028) and were more frequently treated in the day hospital and less in inpatient hospital (41% vs. 52.9%; p=0.037). They furthermore had less treatments in the follow up period (by specialists and alternative practitioners, 53.3 vs. 72.2%; p<0.001) and had to be readmitted less frequently due to a relapse (1.9 vs. 9.6%, p=0.008). They also had a slightly higher level of social functioning in the SOFAS at T0 (10.2 vs. 9.1, p=0.008) and T3 (14.4 vs. 13.6, p=0.014).

**Figure 1:**
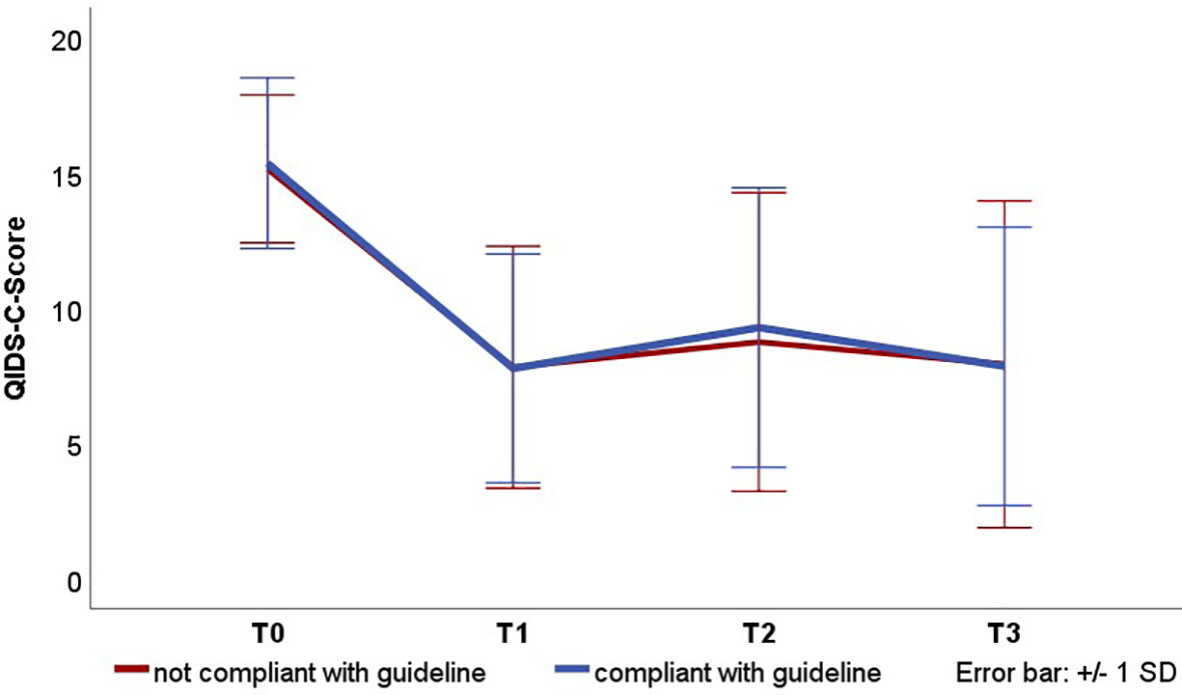
Course of the depressive symptomatology in the QIDS-C scores for therapies complying with the guideline. Presented are the mean values and standard deviations. T0: Admission, T1: Discharge, T2: 3-month follow-up, T3: 12-month follow-up. QIDS-C severity categories: 0–;5 No depression, 6–10 mild, 11–11;15 moderate, 16–20 severe, 21–27 very severe.

For the comparisons of the QIDS-C scores, a repeated measures ANOVA was computed with the two groups as independent variables. The results show a significant effect of the time [F(2.86, 1422) = 326.7, p <.001]. There were no significant differences between the groups according to compliance with the guideline [F (1, 498) = 0,09, p = 0.76] and there was no significant interaction between time and group [F(2.9, 1422) = 0,45, p = 0,71] (see **Figure 1**). Therefore, the hypothesis that patients who are treated in compliance with the guideline have a better outcome has to be rejected.

### Analysis of the Therapy Modality That Is Decisive for Guideline Compliance

**Table 5** illustrates the extent to which each form of therapy influenced the decision that the treatment is in accordance with the guideline.

**Table 5:** Distribution of patients treated in compliance with the guideline according to the respective therapy (N=397 patients treated in compliance with the guideline).

Therapy modality	n	%
In compliance with the guideline, medication only	47	11.8
In compliance with the guideline, psychotherapy only	239	60.2
In compliance with the guideline, combination of medication and psychotherapy	111	28.0
Total	397	100.0

More than 60% of the treatments fulfilled the criterion “compliance with the guideline by outpatient psychotherapy”. More than 25% of treatments matched this criterion by both outpatient psychotherapy as well as anti-depressant medication. Ten percent of the treatments were in accordance with the guideline by anti-depressant medication only.

## Discussion

This study analyzed in how far treatments comply with the guideline recommendations in the year following an inpatient or day-clinic treatment in eight psychosomatic (psychotherapeutic) hospitals in Southern Germany. It was based on the recommendations of the German National “Unipolar Depression” Treatment Guideline (13). Summarizing the most important results, almost 80% of the patients received guideline-compliant follow-up treatment. The majority of the patients continued to receive psychotherapy (approx. 80% of the patients in the overall follow up-period). In the follow-up period of 12 months, 67% of all patients were treated with some form of anti-depressant medication (in a sufficient dosage, but independent from the time criteria of the guideline). For approximately one third of the patients, the dose was changed or a new medication was prescribed in this period of time. The large percentage of patients treated with psychotherapy shows that therapy availability is rather good in Germany.

The data set with 502 patients provides a very large sample for the analyses. The patient clientele is typical for a psychosomatic hospital with a psychotherapeutic focus, with approximately two third of the patients being female and a relatively high level of education (50% with at least 12 years of schooling) compared to two German studies on patients with major depressive disorders in psychiatric hospitals (25, 26). There is another naturalistic follow study on patients with depression who were treated in psychiatric hospitals in Germany (27). But research questions (frequency of relapses), follow up-assessments (every year over 3 years) differed much to our study, so a comparison of the results is not possible.

The initial sample of the INDDEP study consisted of 604 patients. T2- and T3-follow-up data could be assessed for 502 patients (83%), which is in line with other follow-up studies. Nevertheless, the patients who dropped out were younger, had slightly higher depression scores, had less years of school education and were more frequently unemployed. Perhaps their treatment after the hospital stay differs more from that recommended by the guideline, but this is only speculative.

A non-compliance with the guideline was stated if neither an effective anti-depressive medication nor psychotherapy was provided. Whether medication was administered in the hospital was taken into consideration here (corresponding to the acute treatment according to the guideline). All patients received psychotherapy as part of the hospital treatments. Examples for deviations from the guideline recommendations are no psychotherapy or no medication, early discontinuation of the medication or reduction of the medication below the defined threshold.

The hypotheses that patients who are treated in compliance with the guideline have a better outcome had to be rejected. The comparisons of patient characteristics and disorder courses in accordance to the compliance with the guideline yielded only few differences. Patients who received a non-guideline-compliant treatment were somewhat younger, less impaired during the hospitalizations, tended to be treated in day hospital rather than as inpatients, and had a somewhat higher social level of functioning. There were no differences between the two groups in the depression scores in the QIDS-C. One could assume that therapies ended earlier or medications were reduced because these patients were somewhat less impaired and functioned better in social settings. Consequently, “not guideline-compliant” does not automatically mean medically inadequate. It is possible that the patient, the physician or both together agreed to reduce the respective therapy even though it would no longer comply with the proposed guideline. This result fits well with the results of Thompson et al. (7), who in a randomized study were unable to show that training of primary care physicians in guideline therapy resulted in better therapy outcomes.

What do these results imply for health care policies? In Germany, the guidelines are seen as recommendations, but not as fixed rules. The results of this study show, that some deviations from the guideline do not lead to a worse therapy outcome. Thus, utilizing the guidelines as recommendations seems to be quite reasonable. This accounts especially when the guideline recommendations are not based on empirical studies. Concluding, treatment of mental disorders should be understood as a result of an interaction of patient, the physician or psychotherapist, and the therapy course. With this understanding, guidelines can help practitioners to find, together with their patients, the individual treatment decisions.

### Limitations

The disease course and the treatments in the follow-up period were assessed at only two points. Consequently, the LIFE-interviewer had to assess the disease course, the medication and changes of the doses retrospectively over 3 and 9 months. In addition, the LIFE interview was given over the phone. Both of these circumstances might result in a less exact documentation, for example, if a patient could no longer recall when the medication was changed.

The follow up-period with 1 year is rather short. Especially patients with a recurrent depression should be treated with medication for a longer time. This could not be surveyed in this study.

The compliance of patients regarding the medication intake relied on information provided by the patients. At the same time, it is known that medication compliance is often low for anti-depressants (28–30). Reasons for a medication change were also not assessed (e.g. side effects, quick symptom alleviation, interaction with other drugs, rejection by the patients).

The type and duration of psychotherapy is not defined in the guideline. The German guideline recommends psychotherapy between 10 and 36 (individual therapy) sessions, distributed across a period of several months or even several years (6 to 36 months). It was, therefore, relatively easy to meet the outpatient psychotherapy criterion.

Finally, these results are related to the German health care system with its special possibilities like psychotherapeutic inpatient treatments and outpatient psychotherapy. It is not clear, if a study in another country would lead to similar results.

## Data Availability Statement

The datasets generated for this study are available on request to the corresponding author.

## Ethics Statement

The studies involving human participants were reviewed and approved by Ethics Committee of the University of Freiburg (no. 39/11) and the Ethics Committee of the University of Ulm (83/11). The patients/participants provided their written informed consent to participate in this study.

## Author Contributions

LW and JW developed and performed the evaluations on guideline adherence and wrote the manuscript. AZ is the head major researcher of the INDDEP study. AH and ER were responsible for the statistic evaluations and performed them. HW belongs to the directory board of the study. The INDDEP Study Group consists of the directors and researchers of the different sites in this study. All authors contributed to the article and approved the submitted version.

## Funding

The INDDEP study was funded by a grant from the Heidehofstiftung GmbH Stuttgart (No.59055.02.1-4). The Heidehofstiftung had no influence neither on the study design, the collection, analysis and interpretation of the data, nor in writing or submitting this manuscript.

## Conflict of Interest

The authors declare that the research was conducted in the absence of any commercial or financial relationships that could be construed as a potential conflict of interest.
